# Comparative population structure of *Plasmodium malariae *and *Plasmodium falciparum *under different transmission settings in Malawi

**DOI:** 10.1186/1475-2875-10-38

**Published:** 2011-02-11

**Authors:** Marian C Bruce, Allan Macheso, Alex McConnachie, Malcolm E Molyneux

**Affiliations:** 1 Division of Infection and Immunity, Institute of Biomedical and Life Sciences, Level 5, Glasgow Biomedical Research Centre, Glasgow University, 120 University Place, Glasgow University of Glasgow, G12 8TA, UK; 2 Ministry of Health and Population, Government of Malawi, currently at Management Sciences for Health Malawi Programme, Lilongwe, Malawi; 3 Robertson Centre for Biostatistics, Boyd Orr Building, Glasgow University, University Avenue, Glasgow, G12 8QQ, UK; 4 Malawi-Liverpool-Wellcome Trust Clinical Research Programme, College of Medicine, Blantyre, Malawi, and School of Tropical Medicine, University of Liverpool, UK

## Abstract

**Background:**

Described here is the first population genetic study of *Plasmodium malariae*, the causative agent of quartan malaria. Although not as deadly as *Plasmodium falciparum*, *P. malariae *is more common than previously thought, and is frequently in sympatry and co-infection with *P. falciparum*, making its study increasingly important. This study compares the population parameters of the two species in two districts of Malawi with different malaria transmission patterns - one seasonal, one perennial - to explore the effects of transmission on population structures.

**Methods:**

Six species-specific microsatellite markers were used to analyse 257 *P. malariae *samples and 257 *P. falciparum *samples matched for age, gender and village of residence. Allele sizes were scored to within 2 bp for each locus and haplotypes were constructed from dominant alleles in multiple infections. Analysis of multiplicity of infection (MOI), population differentiation, clustering of haplotypes and linkage disequilibrium was performed for both species. Regression analyses were used to determine association of MOI measurements with clinical malaria parameters.

**Results:**

Multiple-genotype infections within each species were common in both districts, accounting for 86.0% of *P. falciparum *and 73.2% of *P. malariae *infections and did not differ significantly with transmission setting. Mean MOI of *P. falciparum *was increased under perennial transmission compared with seasonal (3.14 vs 2.59, p = 0.008) and was greater in children compared with adults. In contrast, *P. malariae *mean MOI was similar between transmission settings (2.12 vs 2.11) and there was no difference between children and adults. Population differentiation showed no significant differences between villages or districts for either species. There was no evidence of geographical clustering of haplotypes. Linkage disequilibrium amongst loci was found only for *P. falciparum *samples from the seasonal transmission setting.

**Conclusions:**

The extent of similarity between *P. falciparum *and *P. malariae *population structure described by the high level of multiple infection, the lack of significant population differentiation or haplotype clustering and lack of linkage disequilibrium is surprising given the differences in the biological features of these species that suggest a reduced potential for out-crossing and transmission in *P. malariae*. The absence of a rise in *P. malariae *MOI with increased transmission or a reduction in MOI with age could be explained by differences in the duration of infection or degree of immunity compared to *P. falciparum*.

## Background

Malaria in humans is caused by four main species of *Plasmodium*: *Plasmodium falciparum*, *Plasmodium vivax*, *Plasmodium malariae *and *Plasmodium ovale. Plasmodium falciparum *is the most prevalent in Africa and the most pathogenic of these, but in most malaria-endemic regions multiple sympatric species are found and co-infection within individual human hosts is common. *Plasmodium vivax *is often found as a co-infection with *P. falciparum *in endemic regions of Asia and South America, but is absent from most of sub-Saharan Africa, where the highest burden of malaria lies. In Africa, *P. malariae *is the species most frequently found in sympatry with *P. falciparum *[1].

Analysis of polymorphic antigen loci and microsatellites in molecular genetic studies of *P. falciparum *have provided great insights into the epidemiology (reviewed in [2,3]) and population biology [4-6] of this parasite. Analysis of such loci have also been used in monitoring the effects of malaria intervention strategies [7-11]. With only a few exceptions [12,13], these studies have been carried out in isolation from data on other sympatric *Plasmodium *species. Population genetic data for *P. malariae *are scarce. Evidence of polymorphism in antigenic loci in *P. malariae *has been obtained using monoclonal antibodies [14] and has been analysed at the genetic level for the circumsporozoite protein [15] and the drug-resistance locus DHFR [16]. The clearest insight into the population genetics of *P. malariae *has been given by microsatellite data which have demonstrated global differences in population diversity and have linked malaria symptoms with a reduction of infection complexity [17].

The importance of *Plasmodium *inter-species interactions to the epidemiology of malaria has been highlighted by a number of studies. Interactions between co-infecting species in humans can modify within-host dynamics [18,19] and alter transmission potential [20]. The effect of mixed species infections on clinical outcome has been described as both beneficial [21] and adverse [22]. Variability in the interactions between species under different transmission intensities, coupled with different sympatric species combinations may contribute to observed differences in the epidemiology and clinical presentations of malaria between endemic regions [23,24]. Evidence to support the notion that interactions can differ between different epidemiological settings has been provided by recent comparative analysis of the clinical impact of multiple infections in three regions of Malawi with differing intensity and seasonality of transmission [25]. This study provides a detailed analysis, at the population genetic level, of *P. falciparum *and *P. malariae *infections reported in the previous study. For the first time the population biology of the two most prevalent, sympatric malaria species in Africa is presented. Polymorphism within microsatellite markers of both *Plasmodium *species was used to determine the population structure of these sympatric parasites within seasonal and perennial transmission regions of Malawi. The study includes infections from asymptomatic carriers of all ages, allowing us to determine how the molecular epidemiology of *P. falciparum *and *P. malariae *changes with age in these populations. Molecular genotyping data from both species has been used to investigate if the clinical severity of malaria is impacted by the complexity of infection and if any such effect is species specific in mixed infections of *P. falciparum *and *P. malariae*.

## Methods

### Study sites and populations

The study was undertaken in villages in Dedza and Mangochi districts of Malawi between 8th March and 7th April 2002. The study sites and the study protocol have been previously described in detail [25]. Briefly, Dedza is a semi-mountainous region in which malaria transmission is restricted to the wet season (November-March). Mangochi district lies 100 km to the east of Dedza, at lower altitude and adjacent to Lakes Malawi and Malombe. Mangochi has perennial malaria transmission. Despite the difference in the epidemiology of the two districts, the overall wet season prevalence of malaria in the population measured using microscopy was remarkably similar, Dedza 23.6%, Mangochi 27.3% [25]. PCR diagnosis increased this to 54.4% and 76.7%, in Dedza and Mangochi, respectively (all *Plasmodium *species). Within Dedza district, there are two transmission zones with higher and lower prevalence, which are likely to result from variation in the intensity of transmission due to altitude. Deaths from malaria are more than three times greater in Mangochi than Dedza [26] and there is a difference in the clinical profile of children admitted for malaria to district hospitals. Children hospitalized with malaria are younger in age and suffer more anaemia in Mangochi than in Dedza [25].

Twenty-four villages (16 from Dedza and 8 from Mangochi) situated within 25 km of the district hospitals were randomly selected for participation in the study. Village sample sizes were based on approximate *P. falciparum *prevalence per district and *P. falciparum*/*P. malariae *ratios to give projected sample sizes of at least 100 *P. malariae *positive individuals per district. All consenting individuals from each village aged >6 months were enrolled in the study. Oral consent for village participation was obtained from village elders and individual written consent was obtained. From a single finger prick, thick and thin blood smears and a filter paper blood sample for molecular analysis were prepared and haemoglobin concentration was measured. Axillary temperature was measured with a digital thermometer and information on the following topics was collected using a questionnaire: age, gender, occupation, religious denomination, occurrence of fever symptoms in previous two weeks, medicine taken in the previous two weeks, overnight stay away from home in previous four weeks, use of bed nets and other anti-malarial prevention measures. Ethical approval for the study was granted by the National Health Sciences Research Committee, Ministry of Health and Population, Government of Malawi and Glasgow University Ethics Committee for Non-clinical Research Involving Human Subjects.

### Detection of *Plasmodium *species and genotyping

Microscopy and PCR diagnosis of *Plasmodium *was carried out as previously described [25]. Briefly, total *Plasmodium *density (all species) was determined by microscopy and an estimated parasite density of 10 parasites per μl of blood was assigned per species detected by PCR in those samples that were microscopy negative. The use of this estimate results in density values below the microscopy sensitivity level of 40 parasites per μl.

Multi-locus genotyping was carried out for the two most prevalent species *P. malariae *and *P. falciparum*. All samples positive by PCR for *P. malariae *were genotyped using six microsatellite markers as previously described [17]. An equal number of samples positive by PCR for *P. falciparum *were selected as comparators. In order that the comparison of *P. malariae *and *P. falciparum *populations were not biased by variation in underlying malariological indices, *P. falciparum *samples were matched to *P. malariae *samples by age of patient (to within one year), gender and village of residence. Where more than one sample met the criteria for matching, a random selection was made from amongst all possible samples. Individuals positive for both *P. malariae *and *P. falciparum *were not excluded from being self-matched. Samples containing *P. ovale *co-infections were not excluded. These infections were ignored for the purposes of all analyses as this species has been shown previously to be not associated with clinical malaria in these samples [25]. *P. falciparum *positive samples were genotyped using 11 tri-nucleotide microsatellite loci [27]. Cycling temperatures, primer concentrations and MgCl_2 _salt concentration of PCR reactions were as described by Anderson *et al *[4] but *Taq *polymerase buffer and enzyme were the same as for *P. malariae *reactions [17].

*Plasmodium malariae *and *P. falciparum *microsatellite PCR products were separated on a 3730 capillary sequencer (Applied Biosystems) following dilution (1:50-1:100) and post-amplification mixing of differently labelled and sized loci. Analysis of electropherograms was carried out using GeneMapper v 3.7 software (Applied Biosystems). Alleles were scored manually and their size measured by comparison with size standard HD400 (Applied Biosystems). To prevent mis-scoring of stutter peaks, secondary alleles were scored only if the peak height was greater than one third of the most intense peak. Whilst ensuring against overestimation of alleles the result of this is to limit the detection of alleles to only those belonging to strains that are present at ~33% or more of the parasite density of the dominant strain. Alleles were binned according to size to within 2 bp. Erythrocytic stages of *Plasmodium *are haploid and, therefore, the presence of multiple alleles within a single sample indicates the presence of multiple genotypes within a species. The number of genotypes per sample, also known as the multiplicity of infection (MOI) [28], was taken to be the greatest number of alleles detected at any single locus for each species. In order to compare the number of genotypes of *P. malariae *and *P. falciparum *per sample, the sensitivity of these measures for each species were balanced by using only a sub-set of *P. falciparum *loci in population analyses. Six out of the 11 available *P. falciparum *loci typed were matched to the six *P. malariae *loci using the number of alleles per locus and heterozygosity (Table 1). Six-locus haplotypes for *P. malariae *and *P. falciparum *samples were constructed using the dominant allele at each locus. The dominant allele in samples containing multiple alleles was defined as that with the highest peak height.

**Table 1:** Matching of six *P. malariae *microsatellite loci to *P. falciparum *microsatellite loci by expected heterozygosity (H_E_) and number of alleles detected

	All data		Dominant only			All data		Dominant only	
***P. malariae*** **locus**	**H_E_**	**Number** **of alleles**	**H_E_**	**Number** **of alleles**	***P. falciparum*** **locus**	**H_E_**	**Number** **of alleles**	**H_E_**	**Number** **of alleles**

Pm09	0.276	11	0.192	8	TAA42	0.551	14	0.442	13
Pm11	0.480	9	0.395	8	377	0.679	10	0.587	8
Pm47	0.516	5	0.471	5	TAA60	0.811	15	0.794	13
Pm34	0.587	13	0.526	11	TAA109	0.841	19	0.820	15
Pm25	0.758	14	0.729	11	TAA81	0.840	13	0.830	10
Pm02	0.862	13	0.849	12	ARA2	0.871	14	0.853	13
					TA1	0.805	29	0.678	25
					2490	0.868	11	0.863	11
					TAA87	0.877	18	0.873	15
					PK2	0.892	19	0.887	17
					Polyα	0.908	21	0.911	19

H_E _values were calculated for all alleles detected, including those from samples with multiple alleles per locus (all data) and from a restricted data set in which only the dominant allele at each locus was used (dominant only). One out of the 12 published *P. falciparum *loci (TA40) [27] was not used as this was found not to amplify.

### Statistical methods

Univariate and bivariate regression models accounting for district (SPSS v10.0, Chicago, USA), were used to determine if the *Plasmodium *genetic variables of species-specific MOI or total *Plasmodium *MOI, were associated with the following clinical outcomes measured during community surveys: haemoglobin level (Hb g/dl), mild anaemia (Hb concentration ≤ 11.0 g/dl), moderate anaemia (Hb concentration ≤ 8.0 g/dl) and fever (axillary temperature ≥ 37.5°C). Linear regression was used for Hb concentration and logistic regression for the binary outcomes of anaemia and fever. Age in years was used as a continuous variable or defined as nine age groups (< 1, 1-4, 5-9, 10-14, 15-19, 20-29, 30-39, 40-49, 50+ years) or individuals were grouped as either children (< 1 to 14 years) or adults (>14 years). Multivariate models were performed using forward inclusion of variables and validated using reverse inclusion. Mann-Whitney U tests were used to compare mean MOI between districts and age groups.

Diversity at microsatellite loci was examined by calculating the expected or "virtual" heterozygosity (*H_E_*) calculated as *H_E _*= [*n*/(*n*-1)][1-Σ*p*_i_^2^] where *n *is the number of samples or alleles detected and *p*_i _is the frequency of the *i*th allele in the population. Expected heterozygosity was calculated using all alleles detected in all samples, including multiple alleles detected in a single sample, and also from a restricted data set in which only the dominant allele at each locus was included. Similarity (*S *) between 6-locus haplotypes was measured using the Jaccard similarity measure or simple matching coefficient [29] and the distance measure 1-*S *was used to cluster samples, using the unweighted arithmetic average. Clustering calculator was used to calculate similarity indices and for the clustering process [30]. Trees were drawn using Treeview v1.6.6 [31].

Population differentiation between districts and villages was assessed using Weir and Cockerham's θ estimator [32] of Wright's Fst statistics, implemented in FSTAT version 2.9.3.2 [33,34]. Between district values were calculated using all six-locus haplotype data whilst between village values were calculated for villages with seven or more *P. malariae *samples. Significance testing was carried out by bootstrapping loci 900 times. To test for linkage disequilibrium between pairs of loci, the log-likelihood ratio G-statistic was calculated from observed data and from randomized data sets permuted 300 times, using FSTAT Version 2.9.3.2 [33,34]. The p-value was estimated as the proportion of statistics from randomized data sets that are larger or equal to the observed. Overall multi-locus linkage disequilibrium amongst 6-locus haploytpes was assessed using a standardized index of association (*I_A_^S^*), implemented in LIAN v3.5 [35,36]. The variance of the number of alleles shared between all pairs of haplotypes in the observed data (*V_D _*) was compared with the variance expected under random association of alleles (*V_E _*) as follows:

(*I_A_^S^*) = (*V_D_/V_E _*-1)(*r*-1), where *r *is the number of loci analysed. Observed data were reshuffled 10,000 times. Separate analyses were carried out for all haplotypes and on a reduced data set where duplicate haplotypes (found in more than one sample) were represented only once, to remove any effect of haplotype sharing. A significance level for p-values of 0.05 was used throughout.

## Results

**Figure 1:**
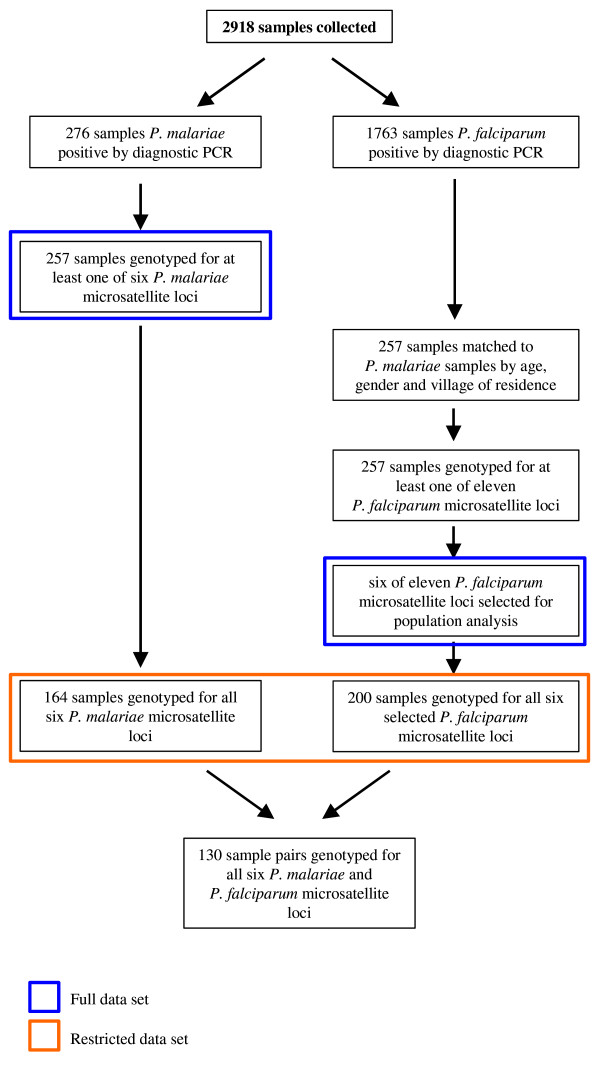
**Flow diagram showing the number of samples collected and analysed for *P. malariae *and *P. falciparum *infections**.

From amongst the 2,918 samples collected from participants resident in Dedza and Mangochi districts of Malawi during the study, 60.4% were *P. falciparum *and 9.4% *P. malariae *positive by diagnostic PCR, Figure 1. Clinical and parasitological differences between the two districts and amongst the three transmission settings within these districts have been described previously [25].

### *Plasmodium malariae *and *P. falciparum *genotyping

In order to compare the population structure of *P. malariae *and *P. falciparum *in Dedza and Mangochi districts we genotyped each species using species-specific microsatellite makers. Multi-locus genotyping was carried out at six *P. malariae *loci [17] and at 11 *P. falciparum *loci [27]. Microsatellite results were obtained from at least one locus in 93% of *P. malariae *infections detected using diagnostic PCR. Complete microsatellite data for all six *P. malariae *loci were obtained from 164 samples. Data from a small subset of these samples (41 out of 257) have been reported previously [17]. An equivalent number of *P. falciparum *samples (n = 257) were genotyped using eleven *P. falciparum *microsatellites. In order to minimize the effect of the epidemiological differences between the two *Plasmodium *species on measurements of population structure, *P. falciparum *samples were matched to *P. malariae *samples by age, gender and village of residence (and hence also transmission region and district), Figure 1.

**Figure 2:**
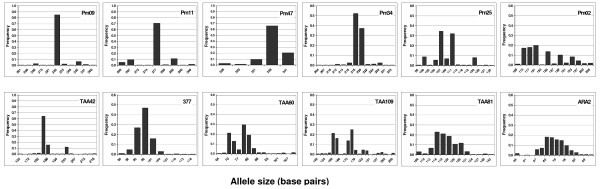
**Allele frequencies of matched *P. malariae *(top) and *P. falciparum *(bottom) loci**. Frequencies shown are for all alleles detected, including all those from samples with multiple alleles per locus.

A sub-set of six *P. falciparum *loci (TAA42, 377, TAA60, TAA109, TAA81, ARA2) were selected for use in population measurements on the basis of allele numbers and heterozygosity being similar to the six *P. malariae *microsatellite loci, Table 1. Allele frequencies for *P. malariae *and the selected *P. falciparum *loci are shown in Figure 2. Complete six-locus *P. falciparum *genotype data were obtained for 78% of selected *P. falciparum *samples. The full two-species data set comprises of 257 sample pairs for which one or more microsatellite loci were typed for both species. A restricted data set comprises of those matched sample pairs for which all six-locus genotypes for both *P. falciparum *and *P. malariae *were available, Figure 1. *P. ovale *was detected by diagnostic PCR in 16.5% of all the samples genotyped for *P. falciparum *and *P. malariae*, but these infections were not genotyped and were ignored for the purposes of all analyses as their presence has been previously shown to be not associated with clinical malaria in these samples [25].

Forty-four percent of genotyped samples were from Mangochi district and 56% from Dedza district. Within Dedza district, more than 80% of samples (both full and restricted data sets) came from the high intensity seasonal transmission region (HIST) rather than the low intensity seasonal transmission region (LIST) [25] owing to the greater prevalence of *P. malariae *in this region.

### Multiplicity of infection

The blood stages of *Plasmodium *are haploid. Therefore, detection of multiple alleles at any locus in one sample indicates the presence of multiple, genetically distinct parasites (genotypes) of that species. The number of different genotypes per sample - MOI [28] - was greater for *P. falciparum *than for *P. malariae*. Maximum MOI in any single *P. malariae *sample was five and for any *P. falciparum *sample was 7 - the greatest overall genotype complexity (combined *P. malariae *and *P. falciparum *MOI) in samples containing co-infections was 9. The percentage of samples that contained multiple genotypes (>1 genotype per species) was high for both species: 59.1% of *P. malariae *and 80.2% of *P. falciparum *samples in the full data set and 73.2% and 86.0%, respectively for the restricted six-locus genotype data set.

The percentage of samples with multiple *P. malariae *genotypes did not differ between Dedza and Mangochi districts (60.0% vs. 58.0%, p = 0.751) nor between children and adults (p = 0.060), Table 2. Despite the epidemiological differences between districts, mean MOI for *P. malariae *(estimated from the restricted data set) was similar in both districts (Dedza, 2.11; Mangochi, 2.12; p = 0.809) and did not differ significantly between children and adults (p = 0.764). Use of the full data set, containing samples with missing data at some loci, did not alter these findings, Table 2.

**Table 2:** *P. malariae *and *P. falciparum *multiplicity of infection (MOI)

	Dedza		Mangochi	
**Number of genotypes**	***P. malariae***	***P. falciparum***	***P. malariae***	***P. falciparum***

1	31 (58)	15 (34)	13 (47)	13 (17)
2	41 (50)	47 (57)	29 (45)	23 (30)
3	23 (26)	24 (31)	14 (16)	20 (25)
4	10 (10)	14 (15)	3 (4)	18 (20)
5	0 (1)	5 (5)	0 (0)	11 (13)
6	0 (0)	3 (3)	0 (0)	6 (6)
7	0 (0)	0 (0)	0 (0)	1 (1)
Total	105 (145)	108 (145)	59 (112)	92 (112)
>1 genotype	74 (87)	93 (111)	46 (65)	79 (95)
Mean MOI children (6 months - 14 years)	2.09 (1.98)	2.56 (2.44)	2.11 (1.85)	3.39 (3.26)
Mean MOI adults (15+ years)	2.17 (1.85)	2.71 (2.22)	2.20 (1.53)	1.62 (1.95)
Total Mean MOI	2.11 (1.94)	2.59 (2.37)	2.12 (1.79)	3.14 (3.04)

Genotypes per sample and mean MOI as determined from six matched microsatellite loci. Values in brackets include samples from the full data set that have missing data at some loci.

The percentage of samples with *P. falciparum *multiple infections was also not significantly different between Mangochi and Dedza (84.8% vs. 76.6%, p = 0.099), but in contrast to *P. malariae*, multiple *P. falciparum *genotypes were more common amongst children than adults (p < 0.01). In contrast to *P. malariae*, mean MOI for *P. falciparum *(estimated from samples with six-locus genotype) was greater in Mangochi compared with Dedza district (Mangochi 3.14; Dedza, 2.59, p = 0.008), Table 2. *P. falciparum *mean MOI was greater in children compared with adults in Mangochi (p < 0.001) but not in Dedza (p = 0.895), Table 2. Children in Mangochi had significantly greater *P. falciparum *mean MOI than children in Dedza (p < 0.001). These findings were not altered by use of the full data set, Table 2. For 59 individual samples containing *P. falciparum*/*P. malariae *co-infections, full six-locus genotypes were available for both species. Amongst these samples the number of *P. malariae *genotypes and the number of *P. falciparum *genotypes were not correlated in either district (Mangochi: n = 20, Spearman rank correlation, 0.101, p = 0.672; Dedza: n = 39, Spearman rank correlation, 0.045, p = 0.786).

Univariate and multivariate regression analyses were carried out separately for *P. malariae *and *P. falciparum *MOI, employing all clinical, parasitological and demographic variables used in previously published epidemiological regression analyses [25]. Levels of clinical malaria and anaemia in this region has been previously described [25] but for the subset of people in this study mild anaemia (Hb ≤ 11.0 g/dl) was detected in 52.7% of individuals, moderate anaemia (Hb ≤ 8.0 g/dl) was seen in 8.4% and fever (temperature ≥ 37.5°C) was observed in 14.3% of cases.

Only *village of residence *and *age *were associated with *P. malariae *MOI and the best multivariate model combined these two variables, Table 3. In a similar analysis for *P. falciparum *the following variables were significantly associated with MOI: *district*, *age*, *age group*, *child/adult group*, *Hb concentration*, *anaemia*, *number of species detected by PCR *and *religion*, Table 4. The best *P. falciparum *multivariate model contained *district *and *child/adult group *variables with a district*child/adult group interaction.

**Table 3:** Univariate and multivariate regression analyses of *P. malariae *MOI

	Univariate Effect			Multivariate Model		
**Variable**	**Beta**	**+/- 95%CI**	**p**	**Beta**	**+/- 95%CI**	**p**
District	-0.143	0.223	0.207	-	-	-
Village*	-	-	**0.037**	-	-	**0.028**
Gender	0.207	0.222	0.068	-	-	-
Age (years)	-0.010	0.009	**0.023**	-0.011	0.009	**0.015**
Age Group*	-	-	0.269	-	-	-
Child/Adult Group	-0.163	0.255	0.209	-	-	-
Fever (Temperature = >37.5°C)	0.082	0.316	0.608	-	-	-
Hb concentration (g/dl)	0.001	0.057	0.972	-	-	-
Anaemia (Hb concentration < = 8 g/dl)	-0.054	0.388	0.784	-	-	-
Treated	0.126	0.239	0.299	-	-	-
Log10 parasite density	-0.098	0.235	0.411	-	-	-
Log10 parasite density estimate including diagnostic PCR data	0.079	0.111	0.165	-	-	-
Number of species detected by PCR	0.156	0.228	0.181	-	-	-
Religion*	-	-	0.162	-	-	-
Fever in last 2 weeks	0.086	0.230	0.465	-	-	-
Anti-malarial taken in last 2 weeks	-0.291	0.804	0.477	-	-	-
Painkiller taken in last 2 weeks	0.061	0.268	0.655	-	-	-
Sleep regularly under bednet	0.118	0.804	0.773	-	-	-
				Model Adjusted R^2 ^= 0.070		

* Individual beta values are not reported for all comparisons between categories.

**Table 4:** Univariate and multivariate regression analyses of *P. falciparum *MOI

	Univariate Effect			Multivariate Model		
**Variable**	**Beta**	**+/- 95%CI**	**p**	**Beta**	**+/- 95%CI**	**p**
District	0.663	0.325	**< 0.001**	2.257	0.981	**< 0.001**
Village*	-	-	0.086	-	-	-
Gender	-0.190	0.335	0.266	-	-	-
Age (years)	-0.022	0.012	**< 0.001**	-	-	-
Age Group*	-	-	**0.010**	-	-	-
Child/Adult Group	-0.700	0.372	**< 0.001**	0.797	0.449	0.318
Fever (Temperature = >37.5°C)	-0.046	0.484	0.853	-	-	-
Hb concentration (g/dl)	-0.130	0.086	**0.003**	-	-	-
Anaemia (Hb concentration < = 8 g/dl)	0.638	0.615	**0.042**	-	-	-
Treated	0.064	0.365	0.731	-	-	-
Log10 parasite density	-0.142	0.315	0.373	-	-	-
Log10 parasite density estimate including diagnostic PCR data	0.016	0.152	0.836	-	-	-
Number of species detected by PCR	0.412	0.248	**0.001**	-	-	-
Religion*	-	-	**0.009**	-	-	-
Fever in last 2 weeks	-0.242	0.347	0.170	-	-	-
Anti-malarial taken in last 2 weeks	-0.509	0.790	0.206	-	-	-
Painkiller taken in last 2 weeks	0.100	0.410	0.633	-	-	-
Sleep regularly under bednet	-0.964	1.020	0.064	-	-	-
Interaction of District and Child/Adult Group	-	-	-	0.338	0.394	**0.006**
				Model Adjusted R^2 ^= 0.111		

To determine if *P. malariae *MOI, *P. falciparum *MOI or combined *P. malariae*-*P. falciparum *MOI were predictive of the clinical outcomes of fever or anaemia we carried out logistic regression. None of the MOI variables was significantly associated with either fever or anaemia.

### Haplotype analysis and population differentiation

Six-locus haplotypes were generated for *P. malariae *and *P. falciparum *from each sample where full genotype data were available. If multiple genotypes were detected in a single sample the dominant allele at each locus was used to generate a single haplotype (see materials and methods). For both *P. malariae *and *P. falciparum *there was a high proportion of unique haplotypes.

From the 164 *P. malariae *samples with six-locus genotype data, 116 different haplotypes were detected. Of these, 89 haplotypes (76.7%) were unique i.e. found only in a single sample and 27 were repeated in more than one sample. The most common haplotype was found in six samples. The expected frequency of each of the repeated haplotypes was calculated using allele frequencies from the restricted data set. The combined observed frequency of repeated haplotypes was in excess of their combined expected frequency assuming random assortment of alleles (p > 0.001).

The proportion of samples with unique *P. malariae *haplotypes was not different between Dedza and Mangochi p = 0.748). Of the 27 haplotypes found in more than one sample (ie not unique), 16 (59.3%) were found in both districts. Of the 23 haplotypes that were found in multiple samples from the same district, 16 (69.6%) were found in different villages. Of the seven haplotypes that were found in multiple samples from the same village, only one haplotype was found in more than one individual from the same household. Even when multiple infections were taken into consideration, out of 49 households in which >1 individual was parasitaemic only eight households (16.3%) included individuals harbouring potentially the same haplotype.

From the 200 *P. falciparum *samples with six-locus genotype data, 193 different haplotypes were detected. Of these, the majority, 186 haplotypes (96.3%), were unique whilst seven were repeated in more than one sample. The seven repeated haplotypes were each found in two samples. As with *P. malariae*, the combined observed frequency of repeated haplotypes was in excess of their combined expected frequency, assuming random assortment of alleles (p > 0.001).

For *P. falciparum*, as for *P. malariae*, the number of samples with unique haplotypes was not different between Dedza and Mangochi (p = 0.417). Of the seven haplotypes found in more than one sample, five (83.3%) were found in only one district and of these, three haplotypes were found in different villages. The two haplotypes found in multiple samples from the same village were detected in individuals living in the same households.

Similarity analysis was used to determine genetic relationships between haplotypes and to examine how these were spatially distributed within villages and districts. No within-district or within-village clustering of *P. malariae *haplotypes was evident. Likewise, no within district clustering was detected for *P. falciparum *haplotypes and there was only limited within-village clustering (6 out of 20 samples from a single village, Mpamanda, Mangochi). Two out of these six samples were from individuals living in the same household. Population sub-structuring was assessed by measuring population differentiation between samples from different villages and districts. Co-ancestry coefficients (θ) were low or negative for both species for all village comparisons and also for between district comparisons, Table 5. None of these comparisons reached significance, therefore there is no evidence to suggest the presence of population differentiation at the village or district level for either *P. malariae *or *P. falciparum*.

**Table 5:** *P. malariae *and *P. falciparum *population differentiation

Species	District	Population	1	2	3	4	5	6	7	8	9
*P. malariae*	Dedza	1 Chinthankwa	-								
		2 Kaphala	-0.021	-							
		3 Kumfunda	0.016	0.004	-						
		4 Makakhula	0.023	0.014	0.015	-					
		5 Thambolagwa	0.003	-0.008	0.059	-0.012	-				
	Mangochi	6 Katema	-0.020	-0.025	0.007	0.014	-0.020	-			
		7 Makawa	-0.037	-0.053	-0.015	0.021	-0.020	-0.072	-		
		8 Matenganya	-0.001	0.001	0.021	0.009	0.036	0.011	-0.049	-	
		9 Mkali A	-0.021	-0.035	-0.083	-0.001	-0.022	-0.038	-0.043	0.008	-
		10 Mpamanda	-0.010	-0.007	0.040	-0.017	0.013	0.009	-0.029	-0.074	0.004
		Between Districts	0.008								
*P. falciparum*	Dedza	1 Chinthankwa	-								
		2 Kaphala	0.013	-							
		3 Kumfunda	-0.034	-0.010	-						
		4 Makakhula	-0.011	-0.002	-0.017	-					
		5 Thambolagwa	0.005	0.000	-0.009	0.036	-				
	Mangochi	6 Katema	0.013	0.018	0.003	-0.011	0.030	-			
		7 Makawa	0.028	0.001	-0.011	0.044	-0.033	0.010	-		
		8 Matenganya	0.013	0.001	-0.004	-0.002	-0.014	-0.018	-0.003	-	
		9 Mkali A	-0.008	-0.004	-0.025	-0.031	0.009	-0.010	0.018	0.002	-
		10 Mpamanda	0.027	0.032	-0.012	0.035	-0.001	0.003	0.008	0.010	0.027
		Between Districts	0.003								

Pairwise between-village and between-district population differentiation for *P. malariae *and *P. falciparum*. Values are Weir and Cockerham's θ estimator of Wright's Fst statistic [32]. Between-district values were calculated using all 6-locus haplotype data whilst between village values were calculated for villages with greater than seven *P. malariae *samples (five villages each from Dedza and Mangochi district; *P. malariae*, Dedza n = 83 and Mangochi n = 45; *P. falciparum*, Dedza n = 89 and Mangochi n = 72). P values did not reach significance for any comparisons.

### Linkage disequilibrium

Pairwise comparisons of the 6 microsatellite loci using re-sampling of the original data provided no evidence for linkage disequilibrium between any pairs of loci for either*P. malariae *or *P. falciparum*. Global multi-locus linkage analysis of haplotypes showed linkage equilibrium among alleles in *P. malariae *haplotypes in both the full data set and in a restricted data set in which duplicate haplotypes were represented only once, Table 5. The same analysis for *P. falciparum *revealed a significant positive association of alleles in the full haplotype data set, which could be attributed to linkage disequilibrium amongst haplotypes in Dedza district only, where transmission is seasonal, Table 6. The value of the standardized index of association (I_A_^S ^) in these analyses were low suggesting limited linkage disequilibrium.

**Table 6:** Multi-locus linkage analysis of *P. malariae *and *P. falciparum *haplotypes

		All data		Unique haplotypes	
		
		**I**_**A**_^**S**^	p-value	**I**_**A**_^**S**^	p-value
***P. malariae***	Both Districts	-0.003	0.650	-0.022	0.994
	Dedza	-0.009	0.765	-0.026	0.986
	Mangochi	-0.003	0.575	-0.032	0.992
***P. falciparum***	Both Districts	0.012	< 0.05	0.009	< 0.05
	Dedza	0.016	< 0.05	0.012	0.055
	Mangochi	0.007	0.181	0.004	0.304

The standardized index of association (*I_A_^S^*) measures linkage disequilibrium by comparing the variance in the number of shared alleles between all pairs of haplotypes with the variance within the randomized data. Unique haplotypes are a subset of the data, in which haplotypes found in more than one sample are represented only once. *P. malariae*, all data: Dedza n = 108, Mangochi n = 59; unique haplotypes: Dedza n = 81, Mangochi n = 35. *P. falciparum*, all data: Dedza n = 108, Mangochi n = 92; unique haplotypes: Dedza n = 106, Mangochi n = 87.

### Human population movement

Movement of the human population could potentially affect parasite population structure by increasing admixture. To assess the extent of human movement participants were asked how often they spent a night away from home and where they visited. Few people (less than 3%) from either district had spent a night away from home in the previous four weeks, and more than 50% of those that did, travelled only within their own district.

## Discussion

*Plasmodium malariae *is the human malaria parasite that gives rise to quartan malaria and the infection is also associated with chronic nephropathy in children. This parasite is distributed worldwide within the tropics and is most commonly found as a co-infection with

*P. falciparum. P. malariae *differs from *P. falciparum *in a number of fundamental biological features. Parasite density is lower, there is a slower growth rate [37], gametocyte production is delayed [38] and duration of infection is longer and often chronic [39]. Until the advent of PCR based diagnostics the low level parasitaemia of *P. malariae *was difficult to detect and its prevalence and contribution to the burden of malaria disease underestimated. The inability to culture the parasite has also hampered its study and comparatively little is known about the epidemiology and population structure of this parasite.

This is the first large scale population genetic study of *P. malariae *and the first to compare the population structure of sympatric malaria species in Africa. This study describes the population structures of *P. falciparum *and *P. malariae*, the most common African malaria co-infections, within the same human population in two sites in rural Malawi. Previously, only the sympatric species combination of *P. falciparum *and *P. vivax *has been studied at the genetic level [13,40]. Caution is needed when interpreting the results of cross-species analyses owing to the many human population, epidemiological and biological differences between species that can confound comparisons. During sample selection we have taken multiple steps to reduce such possible biases and have used genetic markers of similar diversity to analyse the parasite populations. The microsatellite markers we have used for the analysis are not orthologs. Even if orthologous loci for both species were available, this would not guarantee similar allelic diversity or heterozygosity, or immune/evolutionary pressure on such markers in both species. The *P. falciparum *loci used here, although of generally comparable diversity to those of *P. malariae*, are slightly more variable and this has been considered in the interpretation of the results.

A previous study described a lower parasite density and a five-times lower PCR prevalence of *P. malariae *compared with *P. falciparum *among the same samples from the study sites with perennial (Mangochi district) and seasonal (Dedza district) transmission regions [25]. The high proportion of multiple genotypes in *P. falciparum *infections shown in the present study is in line with results from other sites in Africa [4]. However, the high degree of multiple genotype infections also found in *P. malariae *infections was surprising, given the lower prevalence and density (and therefore potential for transmission) of this species, Table 2.

Mean MOI was only marginally greater for *P. falciparum *than *P. malariae *in Dedza (seasonal transmission) and this difference may be in part attributable to the slightly greater diversity of the *P. falciparum *microsatellite markers. The finding that the mean MOI was of a similar value between the two species is surprising, given the dissimilarity in their biology and epidemiology. The lower prevalence and transmission potential of *P. malariae *might be expected to result in lower MOI.

The greater mean *P. falciparum *MOI in Mangochi compared with Dedza is indicative of the perennial nature of transmission in this district and probably results from the cumulative effects of super-infection. However no parallel increase in *P. malariae *MOI was observed - this value being similar to that for Dedza. The absence of an increase in *P. malariae *MOI with increased transmission may be due to differential transmission efficiency of *P. malariae *and therefore insensitivity to this factor, compared with *P. falciparum*. Decreasing *P. falciparum *MOI with age, as seen in Mangochi and in previous studies [41,42], is thought to result from the effects of acquired immunity under intense malaria transmission. The absence of a reduction in *P. malariae *MOI with age may result from limited acquired immunity to this species, which may also contribute to the longevity of the infection.

Regression analyses revealed a negative association of *P. falciparum *MOI with the host's blood Hb concentration as well as associations with age and district of residence. Previous analysis of Hb concentration showed complex relationships with transmission intensity, number of infecting species and age [25]. No relationship between *P. malariae *MOI and Hb concentration was observed. This may be due to the relatively low blood density of this parasite compared with *P. falciparum*. However, the chronic nature of *P. malariae *infections may still have an impact on Hb concentration over the long term. Neither species-specific nor combined MOI measurements were predictive of the malaria symptoms of fever or anaemia. This is in contrast to a previous study in which a reduction of *P. malariae *MOI in symptomatic fever cases compared with asymptomatic ones was observed [17]. In this previous study, symptomatic cases from Gambia and Thailand were compared with asymptomatic ones from Malawi and the lower endemicity of *P. malariae *infections in Gambia and Thailand may underlie this difference.

*Plasmodium falciparum *levels of population differentiation between villages and districts were similar to those found between geographically close African populations described previously [4]. The analyses presented here revealed no indication of population sub-structuring for either *P. malariae *or *P. falciparum *across village or district levels. *P. falciparum *had a higher proportion of unique samples compared with *P. malariae *but most haplotypes (>75%) of both species were unique. A few haplotypes for either species were shared within villages or households but no significant clusters of haplotypes could be detected at village or district levels. The observation that the frequency of repeated *P. malariae *and *P. falciparum *haplotypes exceeds that expected given random assortment of alleles suggests the over-representation in the human population of such halpotypes. This could occur either through sustained transmission via selfing during sexual replication in the mosquito or in the few cases where the same haplotypes are found within members of the same household, through multiple infectious bites from the same mosquito. Together, these lines of evidence suggest that there is sufficient admixture within both species to break down any sub-structuring across the 100 km distance between the districts but that long term persistence of clonal or closely related parasites in a single location is uncommon. Admixture is unlikely to be significantly attributable to human movement as this was low and mostly within district.

Linkage disequilibrium (LD) between *P. falciparum *loci was found only in Dedza district where there is seasonal transmission. Such findings have been found in other seasonally endemic sites in Africa [4]. Absence of *P. falciparum *LD in Mangochi probably results from a higher degree of out-crossing due to greater transmission intensity. In contrast to *P. falciparum*, LD between *P. malariae *loci was not detected despite the lower transmission (and hence lower out-crossing) potential of this species. Alternatively the difference in LD observed between the species may stem from differences in linkage between loci due to the non-orthologous nature of the markers. The chromosomal position of the *P. malariae *markers is not known and so it is not possible to compare the physical linkage of the two sets of markers.

The results of this study, showing high levels of diversity and recombination along with a lack of sub-structuring within *P. malariae *populations is in stark contrast to recent findings for another low prevalence species, *P. ovale*, which is also found across Africa. Population analysis of globally distributed isolates have shown complete segregation amongst six dimorphic loci between classic and variant morphological types of *P. ovale *indicating that these are two non-interbreeding, sympatric species, which have been named *P. ovale curtisi *and *P. ovale wallikeri *respectively [43]. Further investigation using more highly variant microsatellite markers is required to validate the apparent lack of recombination within each new species but current data indicates clonal population structures. Clearly, distinct evolutionary mechanisms are acting across *P. malariae *and *P. ovale *species to result in such contrasting population structures within the same human hosts but the nature of such mechanisms are currently only speculative.

This study took place during the wet season when transmission in both districts is at its peak. A comparison of dry season samples would be useful to determine the differences in the population structure of these co-infections when transmission is either absent or substantially lowered and would shed light on the dynamics of infection over a year.

## Conclusions

This study shows that the population structure of *P. malariae *has similarities to *P. falciparum *that are surprising, considering the biological differences between these species that might favour lower transmission and lower potential for out-crossing of *P. malariae*. These results raise the question of how *P. malariae *achieves such high multiple infection rates or admixture despite its lower prevalence and low parasite densities in human infections. One possibility is that this parasite has evolved a method of increased transmission efficiency in the presence of a co-infecting parasite of another species. A phenomenon of this kind has been observed in murine experimental models of malaria, in which the minority (lower density) species in a mixed infection showed greater transmission potential than the majority species and enhanced transmission compared to single infections [44]. Another explanation is that the chronic nature of *P. malariae *infections may result in more prolonged transmission opportunities than *P. falciparum*.

Despite the similarities in some aspects of *P. malariae *and *P. falciparum *population structure there were also noticeable differences. *P. falciparum *showed changes in MOI with host age and transmission seasonality whereas *P. malariae *did not. These contrasting findings might be a result of differences in the infection or transmission dynamics and immune regulation of the two species.

## Competing interests

The authors declare that they have no competing interests.

## Authors' contributions

MCB conceived and funded the project, led and carried out sample collection in Malawi and performed laboratory and statistical analyses. AM carried out sample collection and led technical teams in Malawi. AMcC advised on statistical analyses and performed some of the analyses. MEM provided logistical support in Malawi, was mentor to MCB and helped to draft the manuscript. All authors read and approved the final manuscript.
